# A rare case of posttraumatic aortic rupture, treated with an endovascular stent graft implantation and complicated with esophageal rupture

**DOI:** 10.1186/s13019-022-01955-y

**Published:** 2022-08-23

**Authors:** Dimitar Kyuchukov, Peyo Simeonov, Gencho Nachev, Magdalena Alexieva, Georgi Yankov

**Affiliations:** 1 Heart Surgery Department, UMBAL “St. Ekaterina”- Medical University of Sofia, 52 A Slaveykov BLVD, 1431 Sofia, Bulgaria; 2 Thoracic Surgery Department, MBALBB “St. Sofia”- Medical University of Sofia, 19 Ivan Gechov BLVD, 1431 Sofia, Bulgaria

**Keywords:** Aortic rupture, Thoracic endovascular aortic repair, Esophageal necrosis, Esophagectomy

## Abstract

**Background:**

Esophageal necrosis and perforation after thoracic endovascular aortic repair (TEVAR) for ruptured traumatic aortic aneurysm is extremely rare. It is difficult to manage, and patients rarely survive without treatment. Although, there is no certain consensus in relation with the optimal treatment we present a subsequent successful management of both life-threatening conditions.

**Case presentation:**

A 52-year-old man experienced a blunt chest trauma after motor vehicle collision with mild symptoms of pain and fractured ribs. On the 12th day he had severe chest pain and computed tomography (CT) revealed a ruptured traumatic thoracic aortic aneurysm with massive mediastinal hematoma. An emergency thoracic endovascular aortic repair (TEVAR) was performed. Several days later the patient developed a fever. CT suspected a pneumomediastinum, a sign of esophageal rupture, but no confirmation from esophagography and esophagoscopy was achieved. Because of deteriorated septic condition, patient was referred for exploratory thoracotomy. The rupture was found and esophagectomy was performed, with an esophagostomy and gastrostomy to enable enteral nutrition. Almost one year after the esophagectomy, gastric conduit reconstruction through the retrosternal route was performed. The patient was still alive and symptom-free more than 1 year after the reconstruction and no infection of the stent graft was observed.

**Conclusion:**

We successfully managed a rare case of esophageal necrosis after TEVAR for ruptured traumatic thoracic aortic aneurysm. It is essential to diagnose the esophageal necrosis at an early stage and provide appropriate treatment to increase survival.

## Background

The incidence of blunt aortic injury (BAI) ranges between 1.5 and 2% of patients involved in motor vehicle collisions, which account for 80% of blunt aortic injuries [1]. BAI is the most common isolated mediastinal injury ranging in severity from an intimal laceration to complete aortic transection. Most patients died immediately from aortic transection. The patients who survive transport to the hospital are those who have sustained contained ruptures or dissections. Undiagnosed injury at the time of presentation significantly increases the chance of fatal complications. Definitive aortic repair is by either open surgery or endovascular treatment. Endovascular aortic repair (TEVAR) is a novel, safe, effective, low-risk minimal invasive therapeutic strategy applied for thoracic aortic aneurysms, dissections and traumatic lesions, even more in elderly, patients in poor condition, high-risk or contraindicated for classical surgical methods. There are many advantages of TEVAR, such as lack of open chest trauma, extracorporal circulation, aortic cross-clamping and single lung ventilation, usage of minimal anticoagulation and less blood loss [2]. Nowadays the endovascular approach by TEVAR is a valuable alternative to open chest surgery with regard to the management of ruptured aneurysm of descending thoracic aorta. The number of patients undergoing TEVAR has increased significantly due to the minimally invasive approach of this technique. Furthermore, a systematic review supports the use of TEVAR, as a first line therapy for BAI [3] and stent-grafting is now the mainstay of management, with success rates ranging from 80 to 100% [4]. TEVAR is also associated with several complications, including paraplegia, renal failure, stroke, post-implantation syndrome, device migration and aortoesophageal fistula (AEF) formation [5]. AEF was defined as any communication between native thoracic aorta or aortic graft and the esophagus. AEF, characterized by the typical Chiari’s triad (pain, sepsis, dysphagia), has been well described in previously published data after the reconstruction of the thoracic aorta [6]. In contrast, there are very few publication for esophageal necrosis with or without perforation described as a rare consequence of TEVAR for both aneurysmal disease and dissection [7–11]. Authors reported that esophageal perforation could be developed between 12 and 2205 days after TEVAR and discussed several potential factors that may result in esophageal necrosis and followed perforation: (1) direct erosion of the stent graft into the esophagus; (2) graft infection; (3) pressure necrosis caused by the self-expanding stent graft; (4) ischemic esophageal necrosis due to disruption of the blood supply to the esophagus; (5) pseudoaneurysm enlargement; (6) mediastinal hematoma reduces intramural arterial blood supply and causing stagnation of venous outflow [3, 8, 9]. As the symptoms of esophageal ischemia and necrosis are usually nonspecific, including pain, sepsis, and dysphagia, this is likely to be an under-recognized complication. Therefore, once transmural necrosis occurs, the prognosis is really poor. According to a paper review only 2 of the 6 published cases with post-TEVAR esophageal necrosis survived [12]. The development of esophageal perforation indicates an infection leading to mediastinitis or sepsis. In any case, perforation becomes the cause of continuous inflammation, and esophagectomy may be necessary for definitive treatment. It is also not sure whether localized infection including stent-graft will improved after esophagectomy. The optimal time of surgical intervention with esophagectomy is before the occurrence of transmural necrosis and in the absence of the subsequent lethal mediastinitis [10]. When mediastinitis is present, the combination of irrigation and drainage and omental translocation are useful surgical interventions [13]. A second staged operation to restore the oesophageal continuity should follow later when the acute infection has resolved and the patient is haemodynamically stable.

## Case presentation

**Fig. 1:**
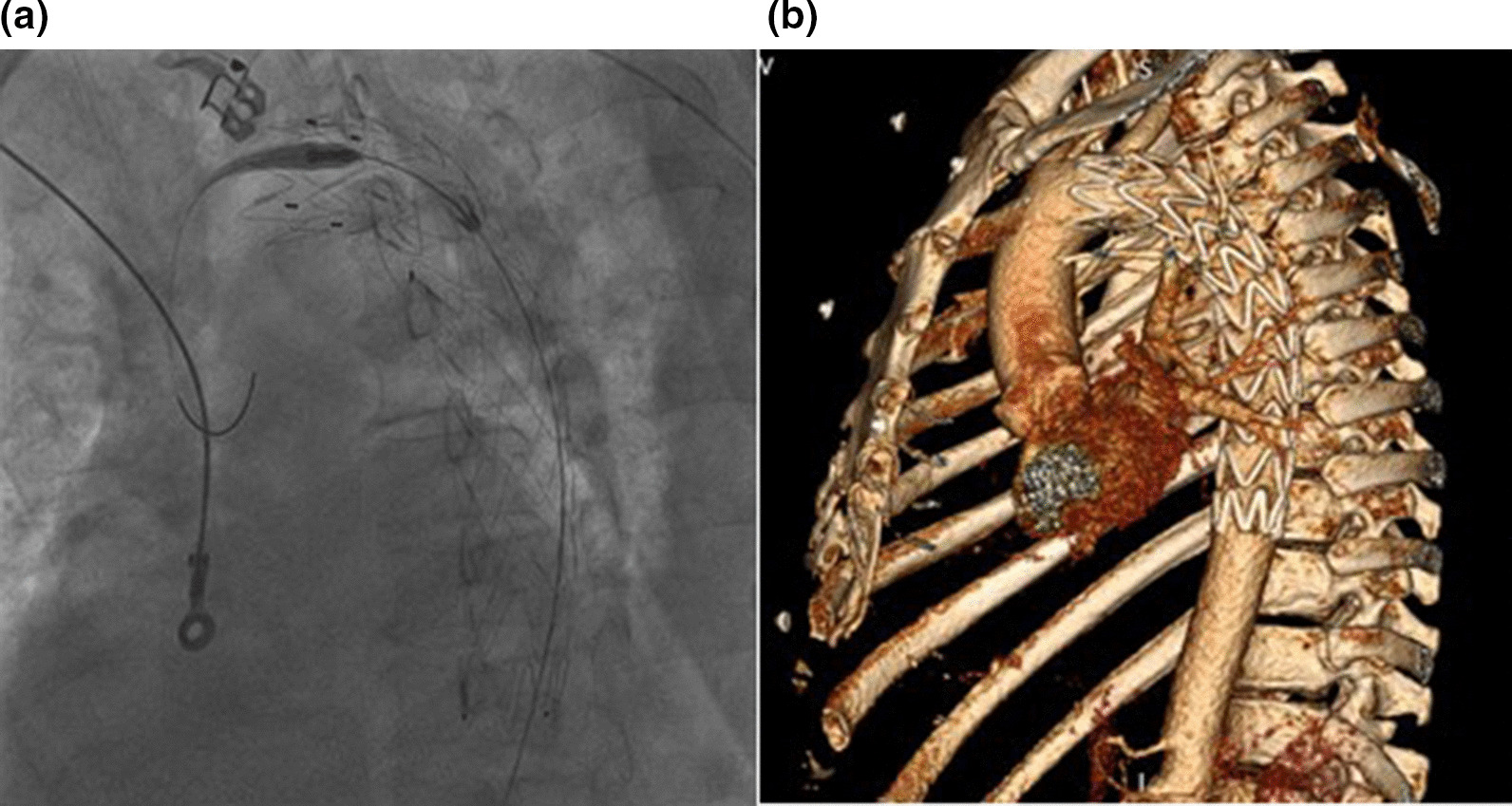
Angiography (**a**) and CT scan reconstruction (**b**), showing a normal position of the aortic stent graft

A 52-year-old man experienced a blunt chest trauma with rib fractures after motor vehicle collision was admitted immediately to a nearby hospital with mild complaints of pain and dyspnea. According to the available medical history, initial chest x-ray showed nothing remarkable except not displaced rib fractures. On the 12th day he had severe chest pain and his condition deteriorated subsequently. An emergent computed tomography (CT) revealed a ruptured traumatic thoracic aortic aneurysm with massive hematoma in the mediastinum. The patient was referred to our cardio-vascular center for urgent treatment. A TEVAR stent graft Valiant Thoracic 32/32/200 mm was successfully deployed (Fig. 1a). The proximal extent of the endograft was ≥ 2 cm distal to the left subclavian artery, which was not covered and ended within the proximal half of the descending thoracic aorta. Even the emergent condition the procedure was uneventful. A left sided chest tube was placed to drain the collected blood. Patient was stabilized and transferred to the intensive care unit. No leakage or residual false lumen flow were verified with control CT (Fig. 1b). The esophageal wall was found thickened and intramural damage could not be rule out, but no mediastinal emphysema or other signs for esophageal rupture observed. On the 7th day after TEVAR patient was discharged and on the 8th became febrile.

**Fig. 2:**
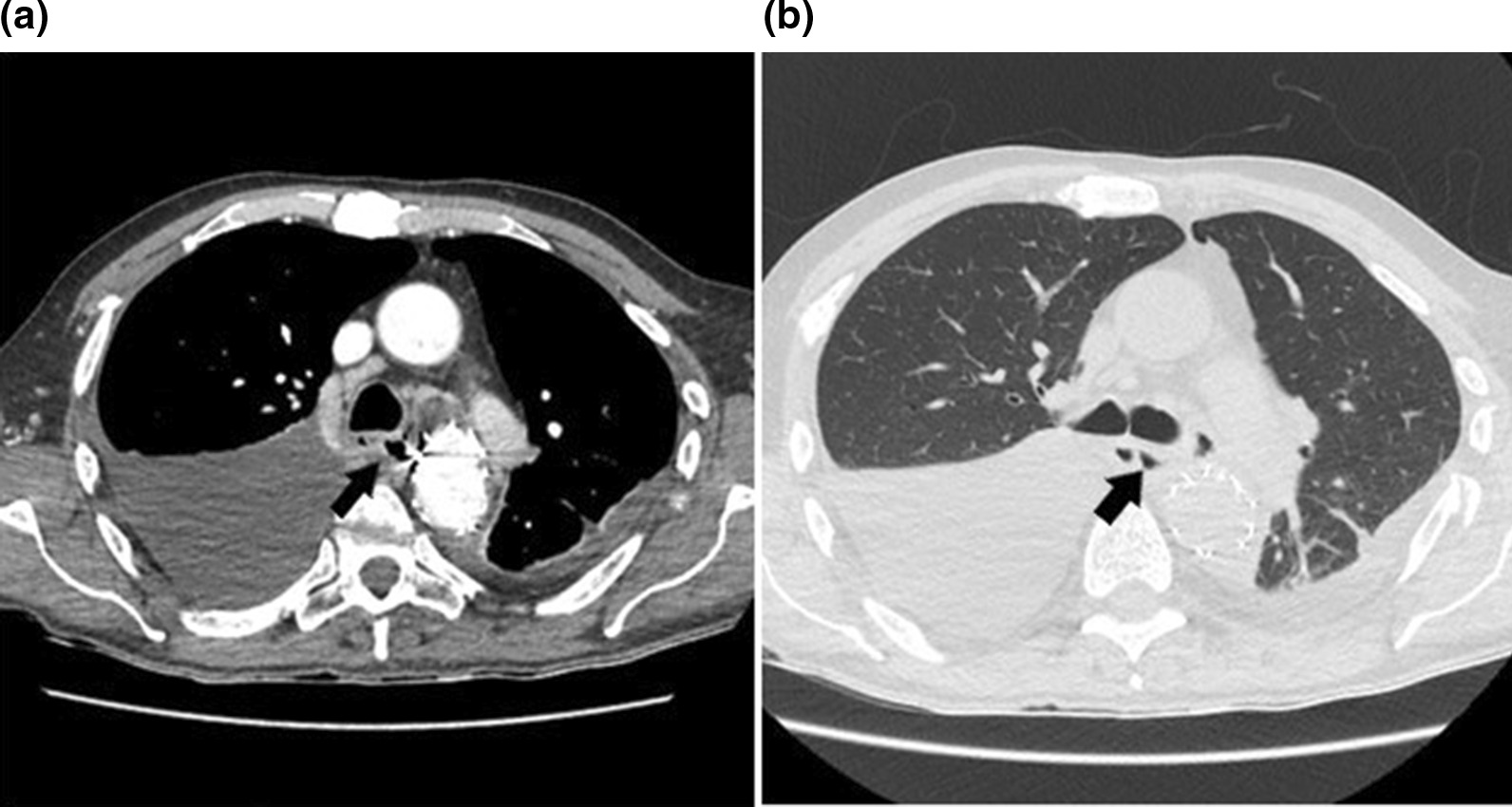
**a**, **b** Axial images of CT scan with intravenous contrast, demonstrating normal positioned aortic stent graft, bilateral pleural effusions and pneumomediastinum, indirect sign of esophageal perforation

A week later he presented with mild dysphagia and intoxication. On admission, the patient was found to be in relatively stable condition with a body temperature of 39 °C, blood pressure of 120/60 mmHg, and a pulse rate of 100 beats/min. Blood tests high inflammation (white blood cell count − 19.6 × 10^9^/L; C-reactive protein- 210 mg/L). Although patient had no hematemesis, nausea or vomiting, a nasogastric tube was placed just in case and total parental nutrition was initiated plus antibiotic treatment with teicoplanin, amikacin, fluconazole. Chest X-ray showed bilateral pleural effusions. CT indicated no endoleak, however there were enlarged posterior mediastinal lymph nodes and heavily dilated stomach, filled with hyperdense fluid, presumably blood and also some concerns of air-fluid levels in mediastinum, around the significantly reduced hematoma (Fig. 2a, b).

**Fig. 3:**
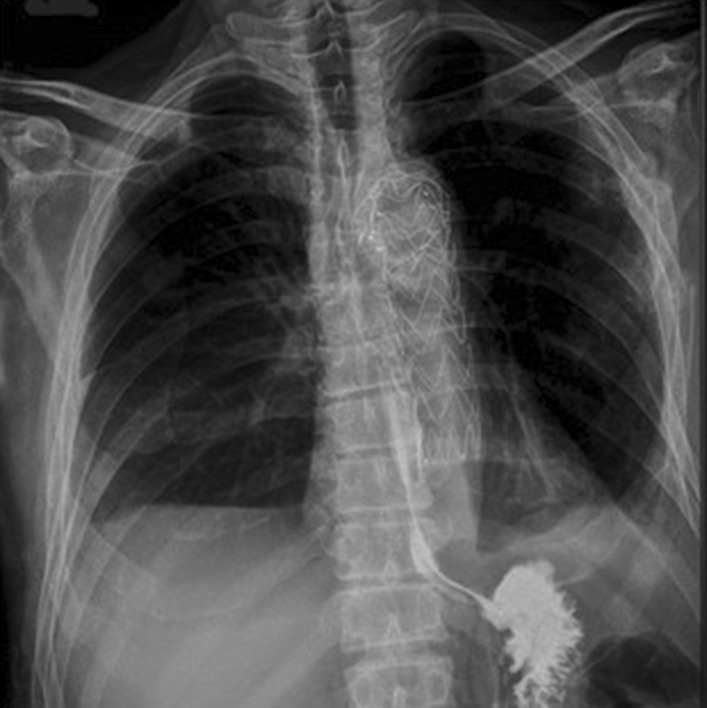
Contrast esophagography, showing no data for mediastinal leakage

To confirm the suggested esophageal perforation barium swallow study was performed, but no evidence of communication was found (Fig. 3).

Endoscopy was also negative for esophageal necrosis either fistula, but a small redness and erosive ambiguous submucosal irregularity was highly suspicious. The persisted fever despite antibiotic administration (changed several times), and also blood tests with high leukocyte count and C-reactive protein level, gathered for discussion an extended multidisciplinary concilium. Taking into account deteriorated septic condition and CT data of probable esophageal rupture, patient was referred for exploratory thoracotomy. Three weeks after the TEVAR a surgical procedure was made with a right thoracotomy in the left lateral position. A 700 ml purulent pleural effusion and plenty of inflammatory adhesions were found. During exploration it was extremely difficult to distinguish aortic wall due to dense adhesions surrounding the esophagus, even though a small perforation of the middle intrathoracic esophagus around 1 cm in diameter was observed. The rupture was in area to the proximal part of aortic stent graft, at the level of proximal anchors, which were in a very close contact to esophageal wall. At least the patient was ultimately diagnosed with esophageal necrosis followed by esophageal perforation and mediastinitis. Esophagectomy and repeated lavage and debridement were proposed. Subtotal esophagectomy along with lateral cervical esophagostomy, omental translocation to buttress the endograft, irrigation and drainage of the mediastinum were performed. The procedure was completed with gastrostomy for enteral alimentation. In the intensive care unit patient remained on assisted mechanical ventilation for 2 days. Enteral nutrition was started on the next day after extubation. Meropenem hydrate (1.5 g/day) and daptomycin (350 mg/day) were administered for 10 days and micafungin (75 mg/day) for 7 days following surgery. The wound gradually healed, and the patient was discharged on 15th day following surgery with persistent esophagostomy and gastrostomy.

**Fig. 4:**
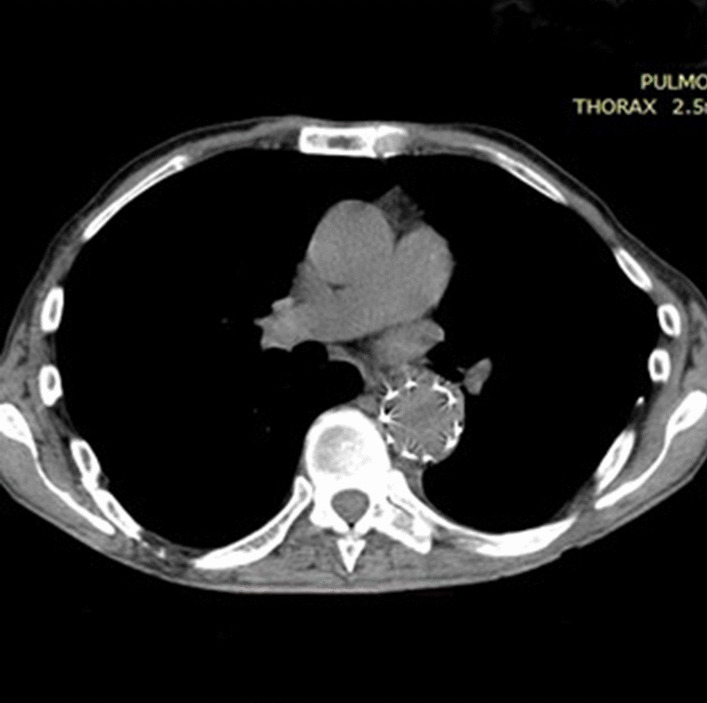
CT scan, illustrating normal position of aortic stent graft and condition after esophagectomy

One year after the esophagectomy the patient was readmitted for restoration continuity of the gastrointestinal tract. The delay was due to the Covid pandemic, but the result wouldn’t be that good if the patient was not so young and compliant. He had no signs of infection during that time. CT scan showed normal position of aortic stent graft (Fig. 4).

**Fig. 5:**
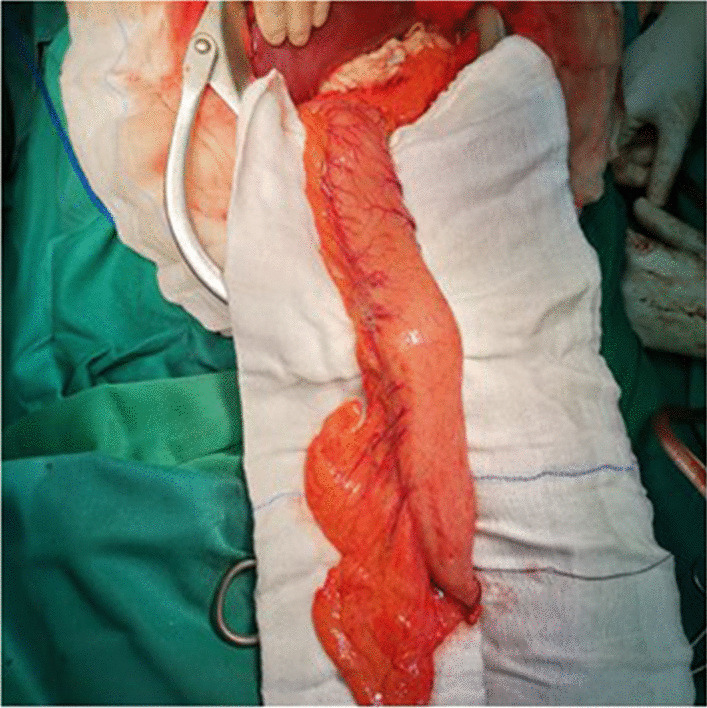
Tailored gastric conduit

We decided to perform gastric conduit reconstruction through the retrosternal route. Under general anesthesia upper midline laparotomy was performed. The gastric conduit (Fig. 5) was made by immobilizing the stomach and forming a gastric tube by resection of the small curvature, during which the left gastroepiploic artery and left gastric arteries were subsequently ligated. The branches of the right gastroepiploic artery and right gastric artery were preserved as feeding vessels.

A retrosternal tunnel was created alternating blunt and sharp dissection. The tunnel was created over the anterior pericardium, meeting the dissection plane from the neck. The conduit was extracted through the tunnel advanced from the cervicotomy. A cervical termino-lateral esophagogastric anastomosis was performed using the hand-sewing technique. The patient recovered uneventfully and discharged on 14th day after the operation. He had close monitoring for the first 6 months and is alive until now (368 days after operative reconstruction).

## Discussion and conclusions

Esophageal necrosis and delayed perforation, secondary to blunt chest trauma and ruptured thoracic aortic aneurysm treated with endograft, is a rare but potentially severe problem. A systemic inflammatory response usually develops rapidly after perforation and overwhelming bacterial mediastinitis may cause circulatory collapse and multiple organ failure with a fatal outcome within a short period of time. We present a case of young man experienced car crash accident with blunt chest trauma complicated with delayed thoracic aortic rupture. Clinical signs are neither sensitive nor specific to diagnose BAI in the hemodynamically stable patient. It was not even thought of an esophageal injury, often difficult to diagnose because of lack of specific symptoms. Although in our case we can discuss a triple mechanism of esophageal injury, necrosis and followed rupture—primary, directly blunt traumatic esophageal injury (unfortunately we didn’t have high quality periprocedural images); secondary, post-aortic rupture esophageal injury (compression by either aortic aneurysm or hematoma from a ruptured aneurysm, extremely elevated mediastinal pressure which cause ischemia as result of reduced intramural arterial blood supply and causing stagnation of venous outflow); thirdly-TEVAR-related causative factors, mentioned above in the background. Traumatic esophageal injuries do not cause a complication leading to death in the early hours in contrast to ruptured aortic aneurysm. Successful TEVAR stent-graft was performed across the reentry of the false lumen and aneurysm was well isolated. The patient was discharged but readmitted a week later. The average time of esophageal necrosis mentioned in publications is the 14th day after a TEVAR procedure, whereas the earliest onset was on the seventh post-TEVAR day [8–11, 14]. Esophageal necrosis should be highly suspected in case of dysphagia within 1 month after a TEVAR procedure, after this period the most probable chronic condition is aorto-esophageal fistula [12]. Differential diagnosis between esophageal necrosis and AEF appears difficult. In our case we didn’t expose the stent graft and a fistula could not be verified. Furthermore, the lack of severe dysphagia or hematemesis, generally vague symptoms and also the transmural esophageal necrosis and rupture that we observed was significantly different from that seen in AEF. Esophageal stent treatment is a temporal solution for AEF, but not for extended necrosis. Very few cases and small series, reported in literature, discussed the management of esophageal necrotic perforation thus there are no accurate and consistent recommendations. The rarity of esophageal necrosis after endovascular grafting along with vague symptoms contribute to delay management. Unfortunately we had to perform explorative thoracotomy to recognize esophageal necrosis and rupture. In our scenario patient had a fever, resistant to antibiotic treatment, but no other specific symptoms. CT scan demonstrated pneumomediastinum, thickened esophageal wall, mediastinal lymphadenopathy, bilateral pleural effusions. The misdiagnosis came from that contrast esophagography did not show any leak and esophagoscopy had minor changes. In this situation the conservative approach with antibiotics, analgetics, parenteral nutrition, protective gastrostomy or enterostomy, proton pump inhibitors, drainage of fluid collections would be an acceptable option. Radical treatment for huge esophageal necrosis/rupture in general, involves exclusion of the infected stent graft and esophageal resection. However, such large surgery is not relevant to most of these patients because of their poor condition. In our case we noted no infection of the stent graft, therefore decided to perform an explorative surgery and after diagnosis adjusment two-stage operation was proposed. The operation of choice was esophagectomy which was the only chance for patient survival [7, 15]. Esophagectomy is a complex surgical procedure most commonly performed for malignancy and sparingly for end-stage benign esophageal diseases. Esophageal exclusion with esophagectomy can be performed as a salvage procedure for the control of mediastinal contamination in cases of uncontrolled esophageal leak or esophageal perforation. According to literature, the optimal time for intervention, was prior to the establishment of transmural necrosis, or else mediastinitis would occur [10]. Despite our delay, esophagectomy debridement, lavage, omentum placement, neck esophagostomy and gastrostomy were successfully performed, giving the patient chance to recover sufficiently and heal the infection. Omentum, as a vascular-rich tissue was used to fill the infected site after esophagectomy and played a major role for inflammation control and buttress the endograft. Reconstructive procedure with gastric conduit was scheduled for six months but Covid pandemic postponed it for another six months. Luckily for us the stent graft did not appear to be infected during that period. Current options for esophageal replacement include the stomach, the right and left colon, and the jejunum. We decided to make a conduit from stomach which we think is the best replacement organ for esophageal reconstruction. The gastric conduit is reliable and the most commonly accepted standard because of its easy access, elasticity, comparably sufficient vascular supply and need for only a single anastomosis. Occasionally, extraanatomic route is required for reconstruction of the esophageal continuity especially in delayed reconstruction. In the majority of cases the preferred route for continuity reconstruction is via posterior mediastinum. In rare circumstances use of the alternative retrosternal route for the conduit placement is required. In cases of long-lasting mediastinal inflammation, debris from aortic aneurysm wall and omentum or caustic esophageal injury, patients develop severe fibrotic reaction and dense adhesions. Retrosternal bypass had been utilized with success in this challenging patient population such as in our situation.

After all, the exact pathophysiology of esophageal rupture in our case remained unclear. Attention has been drawn to three major causes: (1) whether the esophagus was already injured at the very beginning; (2) direct injury of TEVAR proximal hooks, in combination with the (3) extrinsic tension caused by self-expanded graft. However, it is important to recognize the risk of esophageal necrosis following TEVAR for ruptured aneurysm. The prognosis of esophageal perforation after aortic replacement/stenting for thoracic aortic dissection or aneurysm is extremely poor especially in the elderly [7]. We were lucky to deal with a young, relatively healthy man, without comorbidities.

## Conclusion

We successfully managed a rare case of esophageal necrosis after TEVAR for ruptured traumatic thoracic aortic aneurysm. It is essential to diagnose the esophageal necrosis at an early stage and provide appropriate treatment to increase survival.

## Data Availability

Not applicable.

## Abbreviations

AEF: Aortoesophageal fistula
BAI: Blunt aortic injury
CT: Computed tomography
TEVAR: Thoracic endovascular aortic repair
